# Using satellite data on remote transportation of air pollutants for PM_2.5_ prediction in northern Taiwan

**DOI:** 10.1371/journal.pone.0282471

**Published:** 2023-03-10

**Authors:** George William Kibirige, Ming-Chuan Yang, Chao-Lin Liu, Meng Chang Chen

**Affiliations:** 1 Institute of Information Science, Academia Sinica, Taipei, Taiwan; 2 Social Networks and Human Centered Computing Program, Taiwan International Graduate Program, Taipei, Taiwan; 3 National Chengchi University, Taipei, Taiwan; Alagappa University, VIET NAM

## Abstract

Accurate PM_2.5_ prediction is part of the fight against air pollution that helps governments to manage environmental policy. Satellite Remote sensing aerosol optical depth (AOD) processed by The Multi-Angle Implementation of Atmospheric Correlation (MAIAC) algorithm allows us to observe the transportation of remote pollutants between regions. The paper proposes a composite neural network model, the Remote Transported Pollutants (RTP) model, for such long-range pollutant transportation that predicts more accurate local PM_2.5_ concentrations given such satellite data. The proposed RTP model integrates several deep learning components and learns from the heterogeneous features of various domains. We also detected remote transportation pollution events (RTPEs) at two reference sites from the AOD data. Extensive experiments using real-world data show that the proposed RTP model outperforms the base model that does not account for RTPEs by 17%-30%, 23%-26% and 18%-22% and state-of-the-art models that account for RTPEs by 12%-22%, 12%-14%, and 10%-11% at +4h to +24h, +28h to +48 hours, and +52h to +72h hours respectively.

## Introduction

Rapid urban development and industrialization in recent years have increased air pollution, especially PM_2.5_ with an aerodynamic diameter of less than 2.5 micrometers (*μ*m) that cannot be filtered through nasal passages, leading to health problems such as respiratory and cardiac diseases [1–3]. Many nations have built urban stations to monitor the presence of PM_2.5_ in the environment. The resulting datasets can be used to better understand and predict PM_2.5_ [4]. Furthermore, the prediction of PM_2.5_ levels is a difficult problem, as the dispersion of pollutants is heavily dependent on the meteorological characteristics and terrain, in addition to the activities of the inhabitants [5, 6]. The prediction is further complicated by factors such as pollutant migration outside the observed area. Such long-range transport of air pollutants in this work is called Remote Transportation Pollution Events (RTPEs) and relies on wind and other meteorological effects [7]. The aerosol optical depth (AOD) from the Multi-Angle Implementation of Atmospheric Correlation (MAIAC) algorithm is a natural solution to understand air quality in large areas, especially as they have a strong correlation with PM_2.5_ [8, 9]. In this study, we considered pollutants transported from northeast Asia through the East China Sea to Taiwan [10, 11].

Different researchers have used satellite-based AOD measurements to estimate and predict PM_2.5_ due to their high correlation with PM_2.5_ and their large spatial coverage area [12–14]. The AOD of the MAIAC algorithm at 1 km resolution produces better performance than other algorithms with 10 km resolution [2]. Most models use AOD for end-to-end training, where the input to their model is AOD and other related meteorological data, and the output is PM_2.5_ of the same area. In this work, we used MAIAC AOD data from a large remote area as input, while the output is local PM_2.5_ from a part of northern Taiwan. In this work, AOD is not used to directly predict local PM_2.5_, while the proposed model first predicts RTPEs that later with other data are used to predict local PM_2.5_.

In the literature, predictive models are designed using the classical dispersion approach that focuses on identifying the root cause of PM_2.5_ from emissions, chemicals, climatology, or a combination of these factors [15, 16]. For example, the Community Multiscale Air Quality Model (CMAQ) [17] is designed to study air pollution on a global scale. The challenge of this approach includes the failure to detect the complex relationship of features that affect PM_2.5_ [18]. Furthermore, it does not perform well in capturing the spatial and temporal distribution of PM_2.5_ and suffers from high computation costs, especially for a model with complex high-order equations [19].

There are studies that simulate and quantify RTPEs to Taiwan [20–24]. Most of them use trajectory statistics (TS) and chemical transport modeling (CTM) approaches to discover the source of RTPEs. TS uses the frequency of backward trajectories in an area to determine whether pollution is due to remote pollutants. CTM involves a brute-force-based method, which involves two simulations, one without pollutants from the local area and a normal simulation. The difference between these simulations determines the RTPE amounts from the remote area. In this work, we predict RPTEs with the help of several deep neural networks.

The recent development of deep learning in the prediction of air pollution shows the ability to outperform the classic dispersion approach and the statistical approach. Deep learning is based on large historical datasets to capture complex interactions among various features of different datasets. For PM_2.5_ prediction, various deep learning methods involve different machine learning techniques to capture different knowledge from large datasets. Convolutional neural networks (CNN) [25–28] are used to capture spatial knowledge, long short-term memory (LSTM) [27, 28] models are adopted for temporal knowledge, and fully connected neural network (FC) models extract complex interaction between those datasets. Convolutional Long Short-Term Memory (ConvLSTM) is another technique that combines CNN and LSTM to predict PM_2.5_ [26, 29, 30]. However, deep learning with an end-to-end training approach that directly applies the CNN and LSTM components could not perform well for complicated and heterogeneous data [27]. Specifically, the AOD image inputs cause high computation costs and training time that can only obtain a sub-optimal solution. Furthermore, PM_2.5_ prediction becomes more challenging in the long term (for example, for 48 to 72 hours) as there are influences from both known and unknown factors [19]. In this work, we adopted ConvLSTM, CNN and FC to improve the prediction accuracy of PM_2.5_

The philosophy behind deep learning is to produce good results if there is a sufficient training dataset [31]. Due to incomplete data and missing data from different observation stations, the ensemble machine learning approach [18, 32, 33] is used to improve the prediction of PM_2.5_. In an ensemble model, a linear combination of the outputs of different individual deep learning models is used for PM_2.5_ prediction, delivering better results than individual prediction results. These are the popular ensemble machine learning models, AdaBoost (AB) [32], bagging regression (BG), random forest (RF) [34, 35], extreme gradient boosting (XGB) [36–38], and a generalized additive model (GAM) [33, 39]. In this work, we used the composite neural network that outperforms those ensemble models. The composite neural network framework [27, 40] is proposed to resolve complicated applications, such as PM_2.5_ prediction, that connects a collection of pre-trained neural network models to form a large neural network. It is proven that a composite neural network yields greater learning capabilities without the burden of high model training expenses.

Recently, a composite neural network framework that combines different pre-trained deep learning models [27, 40] has been proposed to resolve complicated applications, such as PM_2.5_ prediction. A composite neural network is a collection of pre-trained neural network models that forms a large neural network to yield greater learning capabilities without the burden of high model training expenses. Each pre-trained model utilizes the knowledge from different datasets, and outputs from them are connected into an acyclic tree construction. Later, the outputs are ensembled after constraining the weight of each component into a specific value by using a defined function instead of being ensembled by a weighted average like an ensemble machine learning model

This paper answered two main questions. The first one was about the identification of the occurrence of RTPEs in a local area. The second question was about the incorporation of knowledge about RTPEs to improve the local prediction of PM_2.5_. The questions created three challenges in terms of deep learning design and practice. The first challenge is how to prioritize the factors that influence the capture of remote pollutants, as air quality is affected by multiple factors [9, 28, 41], each with its own spatial and temporal distribution. The next challenge is how to identify factors in the design of a neural network model to capture the complex interactions between them for better PM_2.5_ prediction. The third one is how to fuse and train the proposed neural network model on large heterogeneous datasets for improved efficiency and prediction results.

We addressed the first challenge by considering the AOD data and weather data of remote areas are typically provided in coarse-grained grids. Generally, RTPEs are caused by monsoon and frontal surfaces which are synoptic; therefore, we considered wind speed, direction, and related features. To tackle the second challenge the resulting model [27] was selected as the pre-trained base model, and then including another large deep learning model called spatio-temporal remote information neural network (STRI), it is extended as the proposed remote transported pollutant composite neural network (RTP model). The STRI model incorporates long-range pollutants for PM_2.5_, grasps spatio-temporal features from remote areas and learns the spatial correlation between remote AOD and local PM_2.5_. For the third challenge, we broke the new STRI component into two parts: one for feature extraction and another for prediction. This reduces the number of training parameters and thus the computational cost of the training process with virtually equivalent prediction performance. The four contributions of this work are:

1. The proposed composite neural network RTP model efficiently captures RTPEs and significantly improves PM_2.5_ prediction in comparison with the base model and state-of-the-art models.
2. Addressed challenges using RTPEs as features for local PM_2.5_ prediction. These challenges are addressed in a combined fashion to learn from selected features and models.
3. Developing a classification algorithm to classify RTPEs of two reference sites at different PM_2.5_ levels and increase rates.
4. Applying a composite neural network [42] to develop neural network models incrementally to demonstrate the design rationale and contributions of each component for PM_2.5_ prediction.

## Materials

### Study area

**Fig 1:**
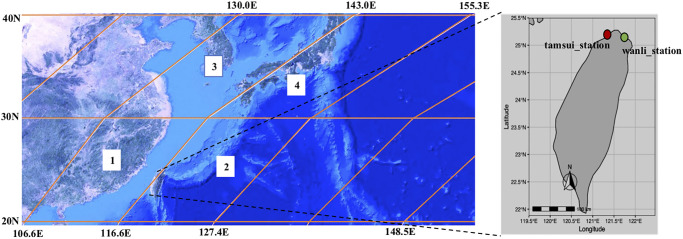
Study area. Left side: Four tiles (adapted from NASA) label 1(*h*28*v*06), 2(*h*29*v*06), 3(*h*28*v*05), 4(*h*29*v*05) with Taiwan in the middle between tiles 1 and 2. Right side: The map of Taiwan after zooming with two stations Wanli (red circle) and Tamsui (green circle).

The area under study comprises of a remote area where we captured RTPEs and a local area (Taipei area) where we performed the prediction of PM_2.5_. The remote area is within the East China Sea consists of four tiles as shown in Fig 1 where each tile covers an area of 1200 x 1200 kilometer square (km^2^). Through that sea the RTPEs from northeastern Asia cross towards Taiwan. The deposition of RTPEs in Taiwan originates from outside Taiwan [20–24]. The Taipei (area = 271.8 km^2^) consists of 18 Environment Protection Administration (EPA) monitor stations where we used two northern shore stations Wanli and Tamsui to demonstrate the existence of RTPEs.

### Dataset

We used the 2014 to 2016 data to evaluate the proposed neural network models. The 2014 and 2015 data were used for training the model and the 2016 data were used for testing. The data for the extended local satellite dataset (ESD) model evaluation were prepared at daily granularity, whereas for the RTP model the data were at hourly granularity.

In both granularities the sample size (N) for the testing dataset varied with the target prediction time, for example the next 1day (+1day) N = 6390 while the next 3day (+3day) N = 12,708. But for the case of + 4*H* hour prediction with *H* ∈ {1, …, 18}, the corresponding number of samples is at most 35120 × *H*. For example, *N* for + 4, + 8, + 64, + 72 hours is 35120, 70160, 558080, and 626400, respectively. Table 1 show sample size (N) for every prediction hour.

**Table 1:** Sample size (N) summary for hourly (+hr) prediction.

Target	N	Target	N	Target	N
+4hr	35,120	+28hr	245,000	+52hr	453,440
+8hr	70,160	+32hr	280,000	+56hr	483,820
+12hr	105,120	+36hr	314,640	+60hr	523,200
+16hr	140,160	+40hr	349,600	+64hr	558,080
+20hr	175,200	+44hr	384,560	+68hr	592,280
+24hr	210,240	+48hr	418,560	+72hr	626,400

#### Observed air quality concentration

We obtained hourly air quality data from the EPA website (data.epa.gov.tw) consisting of PM_10_, PM_2.5_, Carbon monoxide (CO), Nitrogen Oxides (NOx), Ozone (O_3_), and Sulfur dioxide (SO_2_). The Taipei area was divided into grids with total of 1140 (30 × 38) grids cells, thus we used four nearest neighbors (4-NN) method to fill grid cells with empty values. To evaluate ESD, we convert those datasets to daily interval.

#### Meteorological data

The hourly meteorological data were obtained from the Center Weather Bureau (CWB) website (opendata.cwb.gov.tw). Each reading includes wind speed and direction, rainfall, pressure, temperature, and humidity. The data covered 77 grid cells in the Taipei area. Therefore, we used 4-NN to fill those cells without monitor stations. Again, we averaged those datasets into daily reading for ESD evaluation.

#### Remote meteorological data

We used the National Center for Environmental Prediction (NCEP) final (FNL) global analysis data (rda.ucar.edu) covering all over the world. These data are provided over 28 × 28 km^2^ grids every six hours and we converted into hourly interval using linear interpolation. The data includes meteorological features: temperature, pressure, vertical velocity (VVEL), absolute vorticity (ABSV), lifted index, wind speed, and wind direction. The wind speed (*ws*)(denoted as *ws*) and direction(*θ*)(denoted as *θ*) are represented as u and v components, i.e., *ws* × *cos*(*θ*) and *ws* × *sin*(*θ*). The u component is the horizontal speed toward the east (known as Zonal Velocity) and v component is the horizontal wind speed toward the north (known as Meridional Velocity). The *ws* and *θ*, temperature, VVEL and ABSV were considered at pressure levels from 10mb to 1000mb. These data were used for evaluation of the RTP model only.

#### Satellite MAIAC AOD dataset

This is satellite AOD data at a 1 × 1 km^2^ resolution created using the MAIAC algorithm which is updated twice a day and downloaded from the National Aeronautics and Space Administration (NASA) website (ladsweb.modaps.eosdis.nasa.gov). The remote area is covered by four satellite tiles (Fig 1) with the AOD data. The AOD is used to evaluate both RTP and ESD models but the data pre-processing is different for each model.

For ESD evaluation, we used tile 1 and 2 (Fig 1) to fill the AOD data in Taipei area. However, for the missing grids we use the mean of their neighboring grids (3 × 3) to fill their AOD data. For RTP evaluation, we calculated the daily means of AOD value for each grid in all tiles. We assumed the AOD value is the same for the whole day; thus we repeated the same value 24 times to match hourly reading. Furthermore, we also downscaled all tiles to produce a finer spatial resolution. The downscale approach was used on satellite images for precipitation [26] using mean pooling. In this work we use maximum pooling to maintain the distribution of values in each tile. At the end each tile is reduced to spatial dimension of 300 × 300 km^2^. The downscaled tiles match the available memory of graphics processing unit (GPU) and reduce computational cost.

## Method

The description of the methods of the study is based on the composite neural network. The idea is to access or design several pre-trained deep learning models for different tasks and then treat them as the components of the final composite neural networks, ESD and RTP.

### Proposed models

This subsection reports the tasks and the architectures of the following neural network models:STRI model, Base model, Local Satellite Data Model (LSD), RTP model and ESD model.

#### STRI model

**Fig 2:**
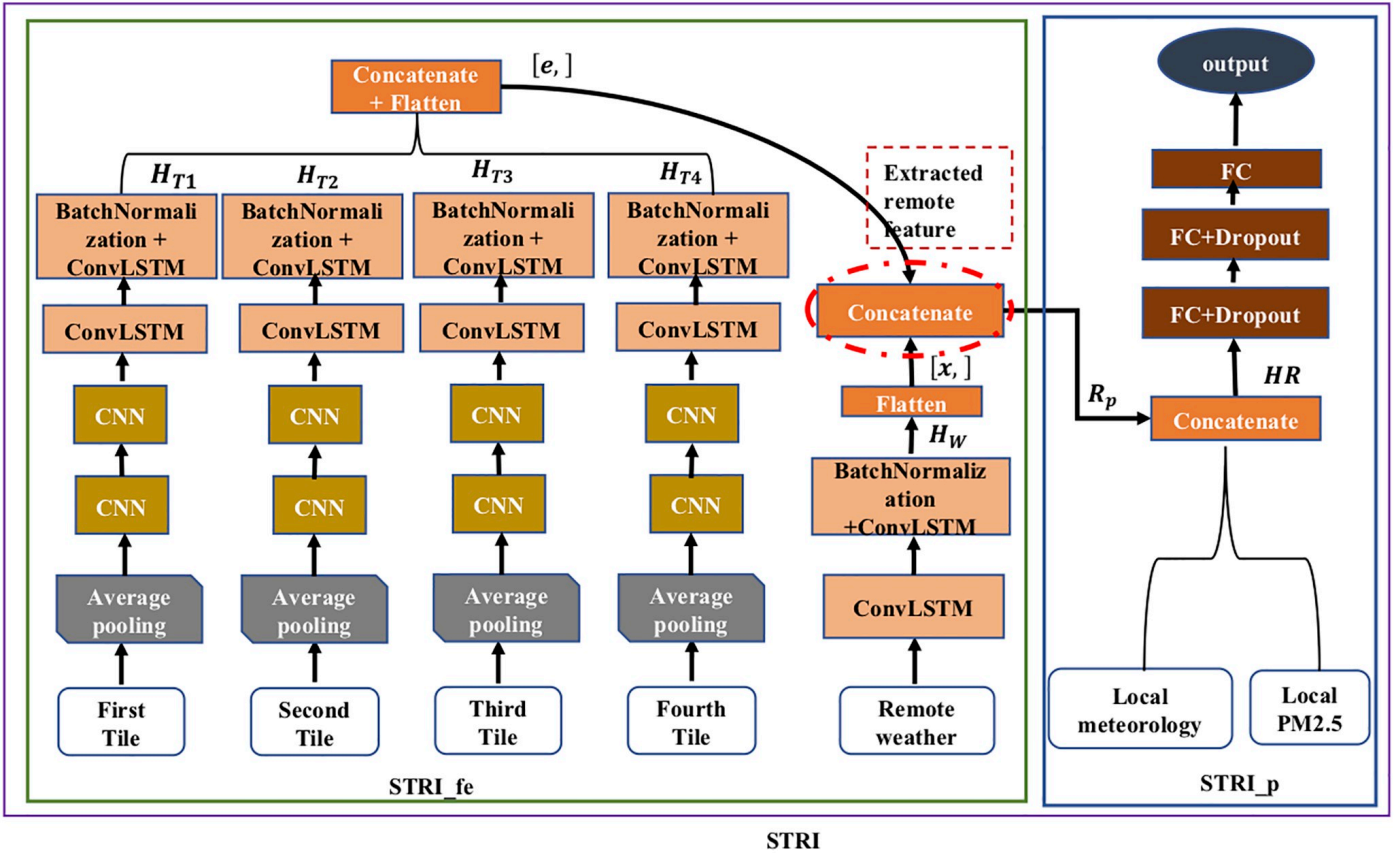
STRI model. Structure of STRI, STRI_fe and STRI_p models, where red dashed circle denotes extraction of spatio-temporal features from remote areas.

As depicted in Fig 2, STRI predicts the PM_2.5_ concentrations of the 18 EPA stations in Taipei using meteorological and AOD features from remote areas with local meteorological features and PM_2.5_ values. Due to the size of both STRI model and its input dataset being larger than the GPU memory limitation and to reduce the computational costs, STRI was divided into the STRI_fe and STRI_p submodels(i.e. components). There are two phases of training, the first trains the STRI_fe model and the second fine-tunes the STRI_p model with fed features from the STRI_fe model.

The inputs of the STRI_fe model are the current four downscaled satellite tiles and remote weather. Each tile is represented by four-dimensional (4d for short) tensor, [*t*, *c*, *w*, *h*] corresponding to time, channel, width, and height. Considering the available memory and computational resources, the model uses average pooling with 3 dimensions (3d) [*c*, *w*, *h*] on each tile along the time axis to reduce their dimension and output *T*_*q*_. The CNN layers receive *T*_*q*_ for capturing spatial correlation, and aggregates information between grid cells. The output from the pooling layer on the 4d tensor is denoted by *P*_*q*_:

$$\begin{array}{c} P_{q}=L\left(\varrho \left(c+b_{i}*v\right)\right) \end{array}$$ (1)

where *L* represented the pooling layer, *c* is the convolutional feature from the convolutional layer, *b* is the additional bias, *v* is a vector with the same size as *c*, and ϱ is an activation function.

$$\begin{array}{c} c=T_{q}*K \end{array}$$ (2)

where *T*_*q*_ was the downsampled AOD data, * represents the convolutional operation, and *K* is the convolutional kernel. To speed up the training process, we applied batch normalization [43] between ConvLSTM layers in the STRI_fe model. The output of ConvLSTM for each tile (*H*_*T*1_, *H*_*T*2_, *H*_*T*3_, *H*_*T*4_) is concatenated and then flattened as a 1 dimension (1d) tensor |*e*|.

On the right hand side of the STRI_fe component, again the ConvLSTM structure with batch normalization is applied to the current remote weather dataset to extract spatio-temporal features, which represent historical weather patterns of wind and other features associated with time and location. Furthermore, the 4d tensor’s output from ConvLSTM, denoted by *H*_*W*_ is flattened as 1d tensor |*x*| which later is merged with |*e*| to form another 1d tensor [*g*] denoted as *R*_*p*_. *R*_*p*_ is the extracted spatio-temporal features of remote pollutants with their corresponding weather features which later is transferred to the STRI_p model after being converted to a 2d tensor.

In the second training phase, the STRI_p model is further refined with the fixed STRI_fe model to reduce the training time, model complexity, and model parameters for improved prediction results. STRI_p receives sequence of *R*_*p*_,local sequences of PM_2.5_ and meteorology data which also include future weather forecasts. Future weather forecast data is included to reflect weather fluctuations, because the current weather is not satisfactory for long-term prediction, i.e., beyond 24 hours [28]. Furthermore, all input features are merged together and form a 2d tensor which is denoted as *HR*. Finally, fully connected (FC) layers are applied to *HR* to learn the complex interaction between features extracted from the remote area and local features and make predictions. More details of STRI model configuration can be found in S1 Table.

#### Base model

The Base model [27] was designed for PM_2.5_ prediction for 18 EPA stations using local influential factors within the Taipei area represented as 30 × 38 = 1140 cells. This model uses 21 features from EPA and 26 features from CWB. Among the 1140 cells, there are 18 EPA stations and 77 CWB stations. This Base model itself is a composite neural network combining six heterogeneous models as its components: one LSTM, two FC (fully connected layer) and three ConvLSTM, where each component has its input data and its expected task. For example, ConvLSTMs extract spatio-temporal knowledge of EPA, CWB and weather forecasting datasets respectively, and two FC are expected to automatically distillate the information from EPA and CWB data. The trained weights of this base are always fixed in our consecutive steps.

**Fig 3:**
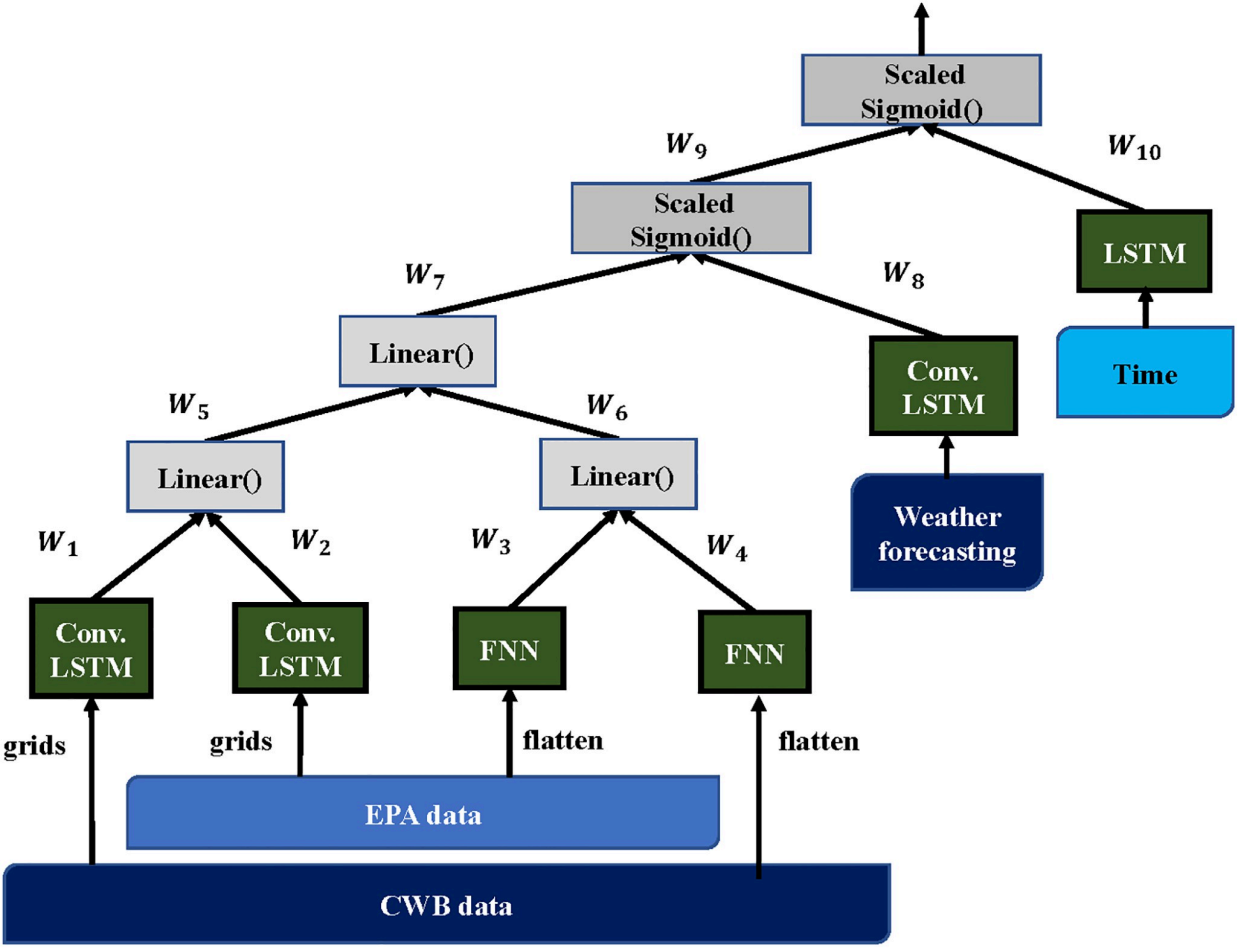
Base model. Structure for Base model for next 24 and 48 hours.

Fig 3 shows the Base model for the next 72-hour prediction. For the next 24-hour and 48-hour predictions, the Base model have the same architecture but different details, such as activation functions and weights (*W*).

#### LSD model

This model only considers local AOD data in the Taipei area, which made it a simpler composite neural network than STRI. We fill the area with AOD PM_2.5_, weather forecast, and meteorological data, all of which are aligned as daily readings and we used them as input to the LSD model.

**Fig 4:**
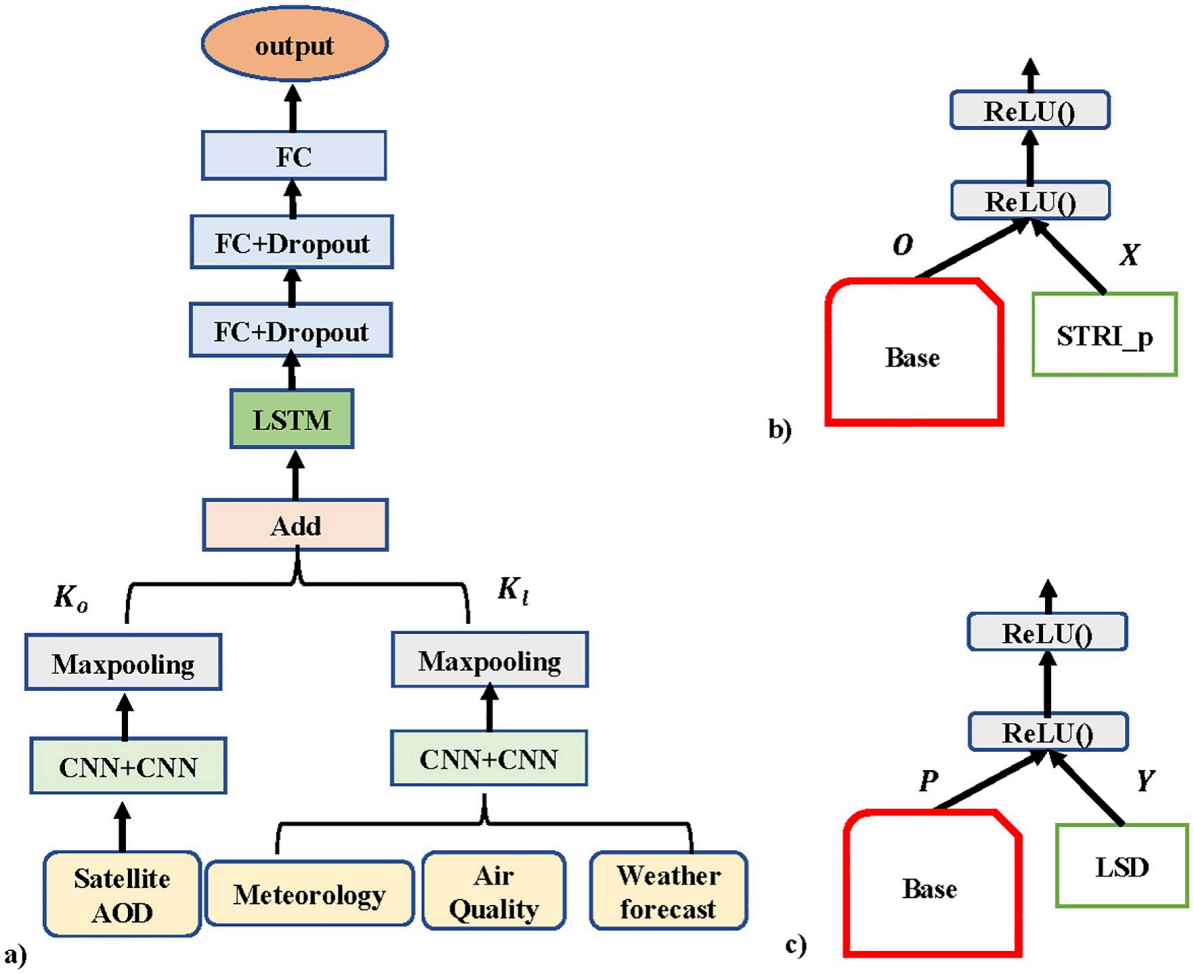
LSD, RTP and ESD models. (a) Structure of LSD model, (b) RTP model, and (c) ESD model.

The LSD model (Fig 4(a)) starts with a series of CNNs on the AOD data to capture the spatial correlation from neighbors along the temporal axis. Then a pooling layer is applied after CNN to reduce the spatial dimensions and aggregate features between the grids and output *K*_*o*_. The model uses the same series of CNN and pooling layers on the current meteorological, air quality, and weather forecast data, and outputs *K*_*l*_. Later, the model concatenated *K*_*o*_ and *K*_*l*_ using an Add layer and LSTM applied to extract temporal related features.

Finally, the FC layer used to learn the interaction and correlation between all features in a nonlinear way [28] and then produces the PM_2.5_ prediction using the final FC layer.

#### RTP model

Consisting of a pre-trained Base model and an STRI_p component, RTP is a composite neural network which handles knowledge from RTPEs. The RTP structure has two components (Fig 4(b)) where each component is trained separately, after which they are used as pre-trained components in the RTP model using a series of ReLU functions (*ReLU*()) for improved overall local PM_2.5_ prediction for the 18 EPA stations.

The RTP model predicts PM_2.5_ concentrations using composite techniques on input from the two components. The PM_2.5_ prediction results from the Base model(O) and STRI_p(X) for 18 stations for the next 4,8 up to 72hour were used as input for RTP. Then RTP predicts PM_2.5_ at the same hour interval for the same stations. The objective here is to improve local PM_2.5_ prediction by accounting for RTPEs.

#### ESD model

We changed the topology of the Base model to the ESD model and apply it for the daily prediction of PM_2.5_. The ESD model with a series of ReLU functions (Fig 4(c)) is composed of the Base model and the LSD model. AOD data in LSD is composed of columnar pollutant measurements as opposed to ground measurements. The difference between the RTP and ESD models is that the ESD has the LSD component with local AOD knowledge to improve daily PM_2.5_ prediction, while the RTP utilizes STRI_p, which learns the remote AOD knowledge.

Given the PM_2.5_ prediction outputs from the Base model(P) and LSD model (Y) for 18 stations for next 1 day to 3 day, then the ESD predicts PM_2.5_ for the same stations at the same daily interval.

## Evaluation

This section reports the experimental environment, the settings and the evaluation for the training of deep learning models.

### Experimental environment and setting

The models were trained on an NVIDIA GPU and implemented on Keras with TensorFlow backend environment. All models were evaluated using root mean square error (RMSE), correlation coefficient (R) and mean bias error (MBE). The RMSE and R evaluate the model predicted values if they represent the true values. Furthermore, MBE estimates the average bias in the prediction. The mathematical equations of those metrics are defined below:

$$\begin{array}{c} RMSE=\sqrt{\frac{1}{n}\sum_{i=1}^{n}{\left(y_{i}-{\hat{y}}_{i}\right)}^{2}} \end{array}$$ (3)

$$\begin{array}{c} R=\frac{\sum_{i=1}^{n}\left(y_{i}-\bar{y}\right)\left(\hat{y_{i}}-\bar{\hat{y}}\right)}{\sqrt{\sum_{i=1}^{n}{\left(y_{i}-\bar{y}\right)}^{2}\sum_{i=1}^{n}{\left(\hat{y_{i}}-\bar{\hat{y}}\right)}^{2}}} \end{array}$$ (4)

$$\begin{array}{c} MBE=\frac{1}{N}\sum_{i=1}^{N}\left({\hat{y}}_{i}\right)-y_{i} \end{array}$$ (5)

where *y* and $\hat{y}$ are true values and predicted value at timestamp respectively. Also $\bar{\hat{y}}=\frac{1}{n}\sum_{i=1}^{n}\hat{y_{i}}$ and *n* is a total number of incident in a sample. In this work we consider the mean of all monitor stations RMSE value.

### Classification of remote pollutants

In this section we answered the first question by classifying PM_2.5_ levels at the Wanli and Tamsui stations in the Taipei region that are affected by RTPEs. The reasons that these two stations are considered the RTPEs indicators include (1) their locations are by the seashore as shown in Fig 1, and (2) their background PM_2.5_ values are stable and relatively low and the occurrence of RTPEs will cause the rise. Therefore, we started by producing PM_2.5_ predictions using the STRI_fe and SRTI_p models without considering the local PM_2.5_ influence factor. In our prediction experiment, we considered only November to May because they are months when RTPEs have the greatest impacts on northern Taiwan [24].

The proposed classification algorithm classifies the PM_2.5_ concentrations for the next 24, 48, and 72 hours (+24h, +48h, +72h) that are affected by RTPEs. Such RTPEs are understood to flow across the eastern China Sea to Taiwan; however, due to variations in wind direction, not all pollutants reach Taiwan. Thus, we seek to ascertain the amount of pollutants reaching Taiwan or the increased concentration caused by the remote pollutants, which corresponds to two conditions of RTPEs. **Condition (1)**: the arrival of such RTPEs raises PM_2.5_ concentrations beyond a certain threshold at these stations. **Condition (2)**: PM_2.5_ concentration increases over two consecutive hours. In particular, Condition (2) assumes if RTPEs arrive in the current hour (t), then the difference between the current PM_2.5_ and that of the previous hour (t-1) must be positive. The RTPEs are said to occur if the peak value simultaneously satisfies both conditions.

#### Classifying remote transportation pollution events

For the two conditions, we created three thresholds for each. That for Condition (1), based on the finding of Chuang et al. [24] showing that the RTPEs in northern Taiwan account for PM_2.5_ concentrations ranging from 31 to 39, we selected 30, 33, and 36 in our experiments. For Condition (2), i.e., the differential threshold (Diff_tshd), the true and predicted PM_2.5_ concentrations were converted to first-order difference vectors, after which the differential thresholds 0.5, 1.0, and 1.5 were set.

**Fig 5:**
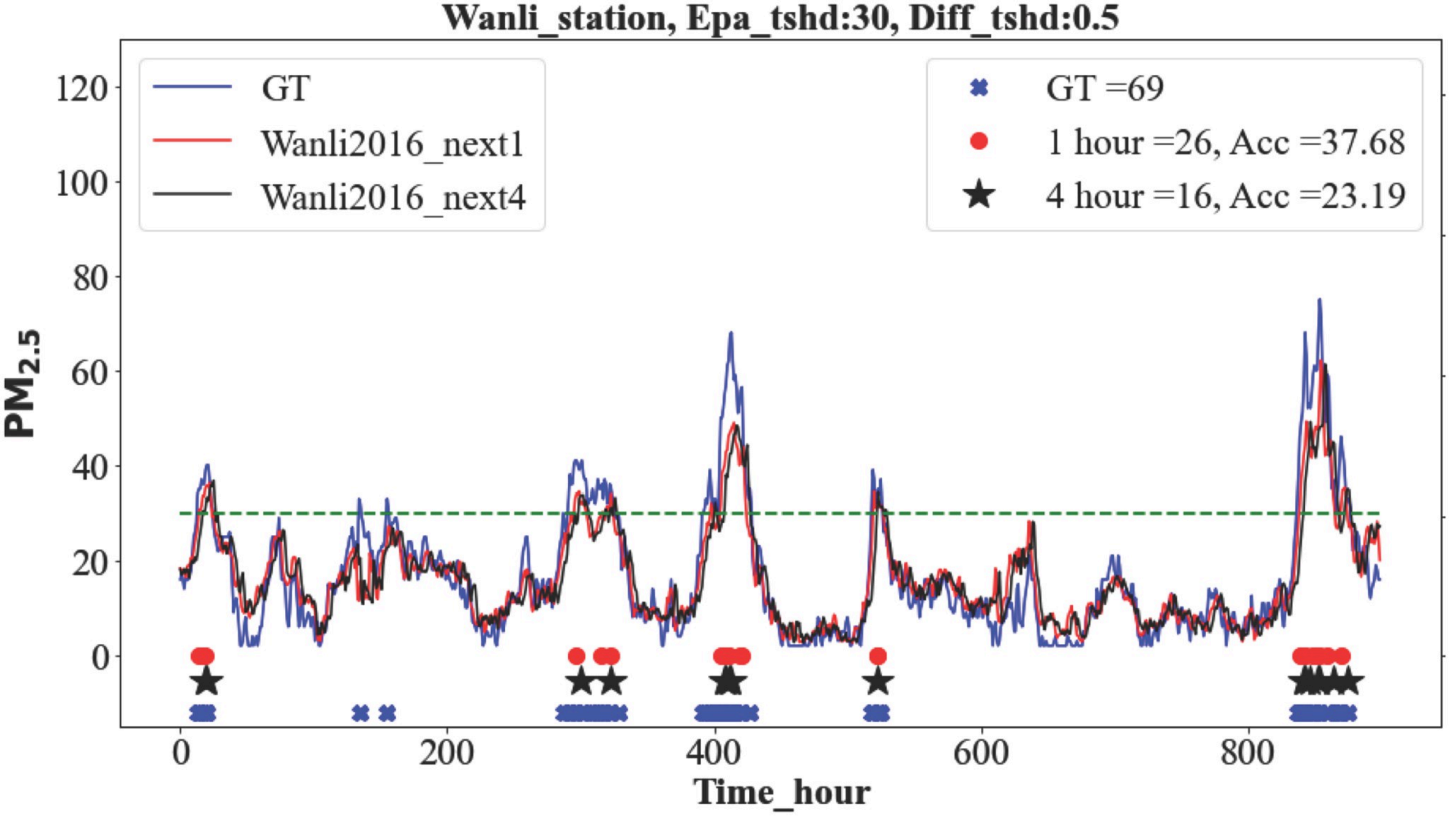
Classify remote pollutant. Prediction of +1h and +4h with ground truth at Wanli station. The dots and stars at the bottom show all peaks that meet both EPA and Differential conditions.

Fig 5 is an example with the ground-truth (GT) and predicted results of the next one hour(+1h) and next four hours(+4h) for the Wanli station. The Epa_tshd and Diff_tshd used in the example are 30 and 0.5, respectively. The green dashed line indicates Epa_tshd, and the colored dots represent the peaks from different predicted hours that exceed the two thresholds. The 26 red dots represent the total number of RTPEs predicted in 1 hour, compared to the 69 ground-truth events. The accuracy of RTPEs detection is thus 37.7%.

Note that RTPEs are defined by conditions that depend on the given Epa_tshd and Diff_tshd thresholds. By definition, a True Positive (TP) RTPEs is when the ground truth and the model prediction are both larger than the Epa_tshd and Diff_tshd. A True Negative (TN) event occurs if neither ground truth nor prediction is larger than the given thresholds. False Positive (FP) and False Negative (FN) events are defined similarly. We used these to calculate the accuracy (A), precision (Pr), recall (R), and F1 score. Specifically, the F1 score is defined as:

$$\begin{array}{c} F1\_score=\frac{2\cdot TP}{(2\cdot TP)+FN+FP} \end{array}$$ (6)

The formulas of the remaining metrics can be found in the deep learning textbook [44]; further classification details are provided in S1 Appendix.

## Results and discussion

### Performance of ESD model

**Fig 6:**
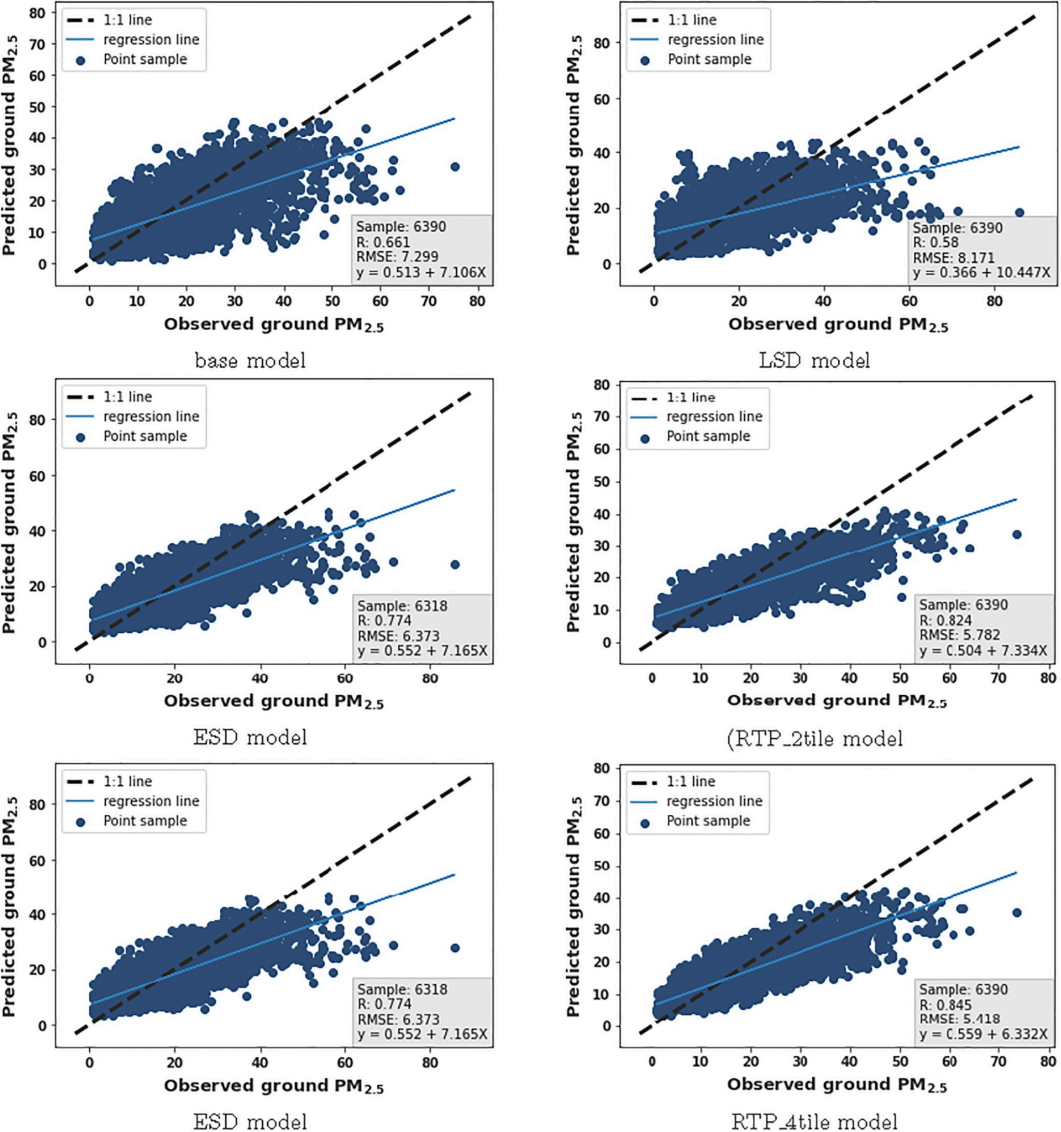
Observed PM_2.5_ vs predicted PM_2.5_ scatter graphs. Plots of association between observed PM_2.5_ vs predicted PM_2.5_ for Base model and proposal models for next 1 day prediction.

In Table 2 we compared the daily PM_2.5_ prediction results of the ESD model with that of its components and the RTP_ktile model (with k = 2,4 representing the number of tiles), where Δ% is the relative improvement in RMSE over the Base model. The Base model outperforms the LSD model for +1day in both R and RMSE, but for +2day and +3day, the LSD component outperforms the Base model due to the application of AOD data. The prediction underestimation (negative) values of the Base model are low compared with LSD however the scatter plots (Fig 6) of observed PM_2.5_ and predicted PM_2.5_ values show few outliers than the LSD model.

**Table 2:** Results for the Base, LSD, ESD and RTP models (the number of samples *N* = 6390, 12744, 18954.).

Target	Model	R	MBE	RMSE	Δ(%)
+1day	Base	0.661	-1.072	7.299	
	LSD	0.580	-0.239	8.170	-11.93
	ESD	0.774	-0.382	6.373	12.68
	RTP_2tile	0.823	-0.975	5.781	18.58
	RTP_4tile	**0.844**	-1.057	**5.418**	**25.77**
+2day	Base	0.363	-0.082	9.802	
	LSD	0.483	-0.760	8.801	10.21
	ESD	0.500	-0.304	8.679	11.45
	RTP_2tile	0.645	-0.114	8.342	14.89
	RTP_4tile	**0.761**	0.185	**6.963**	**28.96**
+3day	Base	0.313	0.123	9.898	
	LSD	0.368	-0.502	9.389	5.14
	ESD	0.386	-0.382	9.240	6.65
	RTP_2tile	0.561	-1.015	8.523	13.89
	RTP_4tile	**0.668**	-0.128	**7.803**	**21.17**

By applying heterogeneous AOD data, ESD model improves its prediction in RMSE by 12.68%, 11.45%, and 6.65% for +1day, +2day, and +3day, respectively. It also shows a better R value than the base model with a prediction bias (underestimation) value of 0.304. The result demonstrates the ESD’s topological changes with the addition of new local AOD knowledge decrease the prediction error between the true and predicted values.

Table 2 also shows that RTP_2tile and RTP_4tile outperformed all those three models for for all target days in RMSE and R. For MBE bias both RTP’s and Base model show positive (overestimation) and negative prediction values at different target day however RTP has few outliers than all the models as shown in scatter plots of observed vs predicted PM_2.5_ (Fig 6). Overall, the results show that the RTP model captures RTPEs from remote AOD data and it helps improve the prediction performance for all days. Regarding the Base model, the RTP_4tile provides the greatest improvement prediction performance in RMSE by 25.77%, 28.96% and 21.17% for +1day to +3day. The RTP_4tile also outperforms RTP_2tile on all three days that the result demonstrates the enlarged remote area will help improve the local prediction of PM_2.5_. This matches with our idea of enlarging the remote area from 2tile to 4tiles to capture more RTPEs.

### Prediction of RTPEs

To answer the first question that we raised in the introduction, i.e., to predict RTPEs, we predicted the local PM_2.5_ for the two stations first using only the local PM_2.5_ and weather as input to the STRI_p model with the extracted spatio-temporal features from remote areas. We predicted the RTPEs by applying the thresholds Diff_tshd and Epa_tshd to the PM_2.5_ predictions. To observe the general performance, we used combinations of various thresholds that Diff_tshd = 30, 33, 36 and Epa_tshd = 0.5, 1.0, 1.5. For example, we combined Epa_tshd = 0.5 with Diff_tshd = 30, 33, 36 and also combined Epa_tshd = 1.0 with Diff_tshd = 30, 33, 36 as it shown in both Tables 3 and 4. Tables 3–5 shows the classification results in terms of accuracy (A), precision (Pr),recall (R), and F1 score (F1). The first column indicates the data used, for instance, “P” represents the local PM_2.5_ values, “EP” represents the remote spatio-temporal features from four tiles, and “W” represents the local weather features.

**Table 3:** Classification results with *Diff_tshd = 0.5*.

Wanli Station												
*Epa_tshd*	30											
Hour	+24h				+48h				+72h			
	A	Pr	R	F1	A	Pr	R	F1	A	Pr	R	F1
P	0.44	0.20	0.32	0.25	0.19	0.10	0.17	0.12	0.08	0.06	0.08	0.07
P+EP	0.71	0.20	0.43	0.28	0.26	0.12	0.22	0.16	0.06	0.05	0.06	0.06
P+EP+W	**0.72**	**0.23**	**0.44**	**0.30**	**0.33**	**0.14**	**0.26**	**0.18**	**0.21**	**0.12**	**0.18**	**0.14**
*Epa_tshd*	33											
P	0.37	0.22	0.29	0.25	0.14	0.10	0.14	0.12	0.02	0.03	0.03	0.03
P+EP	0.61	0.22	0.41	0.29	0.10	0.08	0.10	0.09	0.02	0.02	0.02	0.02
P+EP+W	**0.62**	**0.24**	**0.41**	**0.30**	**0.16**	**0.12**	**0.15**	**0.14**	**0.08**	**0.08**	**0.08**	**0.08**
*Epa_tshd*	36											
P	0.30	0.20	0.25	0.22	**0.11**	**0.10**	**0.11**	**0.10**	0.02	0.03	0.02	0.02
P+EP	0.45	0.22	0.34	0.27	0.02	0.06	0.02	0.03	0.01	0.03	0.01	0.02
P+EP+W	**0.46**	**0.24**	**0.34**	**0.28**	0.06	0.08	0.06	0.07	**0.03**	**0.04**	**0.03**	**0.04**
Tamsui Station												
*Epa_tshd*	30											
P	0.50	0.25	0.35	0.29	0.32	0.18	0.26	0.21	0.19	0.16	0.17	0.16
P+EP	**0.66**	0.28	0.42	0.34	0.32	0.20	0.26	0.22	0.19	0.16	0.17	0.16
P+EP+W	0.63	**0.29**	**0.41**	**0.34**	**0.41**	**0.22**	**0.31**	**0.25**	**0.24**	**0.17**	**0.20**	**0.19**
*Epa_tshd*	33											
P	0.38	0.23	0.29	0.26	**0.20**	0.13	**0.17**	0.15	0.09	0.10	0.08	0.09
P+EP	**0.54**	0.27	**0.36**	0.31	0.15	0.15	0.14	0.14	0.11	0.14	0.10	0.12
P+EP+W	0.51	**0.29**	0.35	**0.32**	0.18	**0.17**	0.16	**0.17**	**0.13**	**0.17**	**0.12**	**0.14**
*Epa_tshd*	36											
P	0.29	0.22	0.24	0.23	0.08	0.09	**0.08**	0.09	0.02	0.05	0.02	0.03
P+EP	**0.38**	0.27	**0.30**	0.28	0.03	0.05	0.04	0.04	0.05	0.09	0.05	0.06
P+EP+W	0.37	**0.30**	0.29	**0.29**	0.07	**0.15**	**0.08**	**0.10**	**0.06**	**0.13**	**0.06**	**0.08**

**Table 4:** Classification results with *Diff_tshd = 1.0*.

Wanli Station												
*Epa_tshd*	30											
Hour	+24h				+48h				+72h			
	A	Pr	R	F1	A	Pr	R	F1	A	Pr	R	F1
P	0.35	0.17	0.32	0.22	0.14	0.07	0.16	0.10	0.05	0.04	0.07	0.05
P+EP	**0.56**	0.16	**0.44**	0.24	0.19	0.10	0.20	0.13	0.05	0.05	0.06	0.05
P+EP+W	0.54	**0.18**	0.43	**0.25**	**0.27**	**0.12**	**0.27**	**0.16**	**0.17**	**0.10**	**0.19**	**0.13**
*Epa_tshd*	33											
P	0.31	0.18	0.30	0.23	0.10	0.07	0.12	0.09	0.01	0.02	0.02	0.02
P+EP	**0.51**	0.19	**0.41**	0.26	0.07	0.07	0.09	0.07	0.02	0.03	0.02	0.02
P+EP+W	0.49	**0.20**	0.40	**0.26**	**0.14**	**0.10**	**0.16**	**0.13**	**0.07**	**0.08**	**0.09**	**0.08**
*Epa_tshd*	36											
P	0.24	0.15	0.25	0.19	**0.09**	**0.08**	**0.11**	**0.09**	0.01	0.01	0.01	0.01
P+EP	**0.37**	0.19	**0.34**	0.24	0.02	0.05	0.02	0.03	0.01	0.03	0.01	0.02
P+EP+W	**0.37**	**0.20**	**0.34**	**0.25**	0.05	0.08	0.07	0.07	**0.03**	**0.04**	**0.04**	**0.04**
Tamsui Station												
*Epa_tshd*	30											
P	0.40	0.20 0	.35	0.25	0.26	0.15	0.26	0.19	0.15	0.12	0.16	0.14
P+EP	**0.54**	0.23	**0.42**	0.30	0.25	0.17	0.25	0.20	0.13	0.14	0.15	0.14
P+EP+W	0.52	**0.25**	0.41	**0.31**	**0.33**	**0.18**	**0.30**	**0.22**	**0.19**	**0.14**	**0.20**	**0.17**
*Epa_tshd*	33											
P	0.32	0.19	0.28	0.23	**0.17**	0.12	**0.17**	0.14	0.07	0.08	0.08	0.08
P+EP	**0.44**	0.22	**0.35**	0.27	0.11	0.12	0.12	0.13	0.08	0.12	0.09	0.10
P+EP+W	0.42	**0.25**	0.34	**0.29**	0.15	**0.14**	0.15	**0.15**	**0.11**	**0.15**	**0.12**	**0.13**
*Epa_tshd*	36											
P	0.23	0.17	0.23	0.20	0.06	0.06	0.07	0.07	0.02	0.05	0.03	0.04
P+EP	**0.31**	0.22	**0.29**	0.25	0.03	0.05	0.04	0.04	0.04	0.09	0.05	0.06
P+EP+W	0.30	**0.24**	0.28	**0.26**	**0.06**	**0.12**	**0.07**	**0.09**	**0.05**	**0.13**	**0.06**	**0.09**

**Table 5:** Classification results with *Diff_tshd = 1.5*.

Wanli Station												
*Epa_tshd*	30											
Hour	+24h				+48h				+72h			
	A	Pr	R	F1	A	Pr	R	F1	A	Pr	R	F1
P	0.44	0.17	0.32	0.22	0.17	0.07	0.16	0.10	0.06	0.04	0.06	0.05
P+EP	**0.68**	0.17	**0.43**	0.24	0.20	0.09	0.18	0.12	0.06	0.05	0.06	0.05
P+EP+W	0.66	**0.18**	0.42	**0.26**	**0.33**	**0.12**	**0.26**	**0.16**	**0.20**	**0.10**	**0.18**	**0.13**
*Epa_tshd*	33											
P	0.39	0.18	0.30	0.23	0.10	0.06	0.10	0.08	0.02	0.02	0.07	0.016
P+EP	**0.63**	0.20	**0.41**	0.26	0.08	0.07	0.08	0.07	0.02	0.03	0.02	0.03
P+EP+W	0.59	**0.20**	0.39	**0.27**	**0.16**	**0.10**	**0.15**	**0.12**	**0.09**	**0.08**	**0.09**	**0.08**
*Epa_tshd*	36											
P	0.29	0.15	0.25	0.19	**0.09**	0.07	**0.09**	**0.08**	0.01	0.01	0.01	0.01
P+EP	0.43	0.19	**0.33**	0.24	0.02	0.05	0.02	0.03	0.01	0.03	0.01	0.02
P+EP+W	**0.44**	**0.21**	**0.33**	**0.26**	0.06	**0.08**	0.07	0.07	**0.03**	**0.04**	**0.04**	**0.04**
Tamsui Station												
*Epa_tshd*	30											
P	0.46	0.20	0.34	0.25	0.30	0.14	0.25	0.18	0.16	0.11	0.15	0.13
P+EP	**0.63**	0.24	**0.41**	0.30	0.29	0.16	0.24	0.19	0.15	0.14	0.14	0.14
P+EP+W	0.61	**0.25**	0.40	**0.31**	**0.38**	**0.18**	**0.30**	**0.22**	**0.22**	**0.15**	**0.19**	**0.17**
*Epa_tshd*	33											
P	0.37	0.20	0.28	0.23	**0.18**	0.11	**0.16**	0.13	0.08	0.08	0.07	0.08
P+EP	**0.49**	0.22	**0.34**	0.27	0.10	0.11	0.10	0.10	0.09	0.13	0.09	0.10
P+EP+W	**0.49**	**0.25**	**0.34**	**0.29**	0.16	**0.14**	0.15	**0.14**	**0.13**	**0.15**	**0.12**	**0.13**
*Epa_tshd*	36											
P	0.27	0.18	0.23	0.20	0.05	0.06	0.06	0.06	0.03	0.05	0.03	0.04
P+EP	**0.32**	0.22	**0.27**	0.24	0.03	0.04	0.03	0.04	0.05	0.09	0.05	0.07
P+EP+W	**0.32**	**0.24**	**0.27**	**0.25**	**0.07**	**0.12**	**0.07**	**0.09**	**0.06**	**0.13**	**0.07**	**0.09**

We first noted the increases in accuracy and other metrics for many forecasts when EP and W are added to the model, which demonstrates the contribution of RTPEs to increasing PM_2.5_ concentrations at the stations. A similar trend is observed for the Epa_tshd, although the increases in accuracy are lower. For example, for the next 24hour (+24h in short) predictions for the Wanli station with a Diff_tshd of 0.5, the accuracy is 0.72, 0.62, and 0.46 for Epa_tshd thresholds of 30, 33, and 36, respectively.

This shows that as the threshold of PM_2.5_ increases, the prediction of PM_2.5_ tends to be conservative and cannot follow the PM_2.5_ increase resulting in low accuracy. In terms of precision and recall, the highest recall of 0.44 is observed at Wanli whereas for Tamsui, the highest is 0.41, both at thresholds of 0.5 (Diff_tshd) and 30 (EPA_tshd) for +24h. For precision, the highest score is 0.24 and 0.30 at thresholds of 0.5 (36) for the Wanli and Tamsui stations, respectively. Overall, these results demonstrate the effects of the STRI model on the RTPEs prediction of these two stations in northern Taiwan.

### Performance of RTP model

In this section we answer the second question about improving local PM_2.5_ predictions using knowledge about RTPEs. We discussed the results of different training approaches for the RTP components, knowledge captured from RTPEs, and the results of RTP models in comparison to other models.

**Fig 7:**
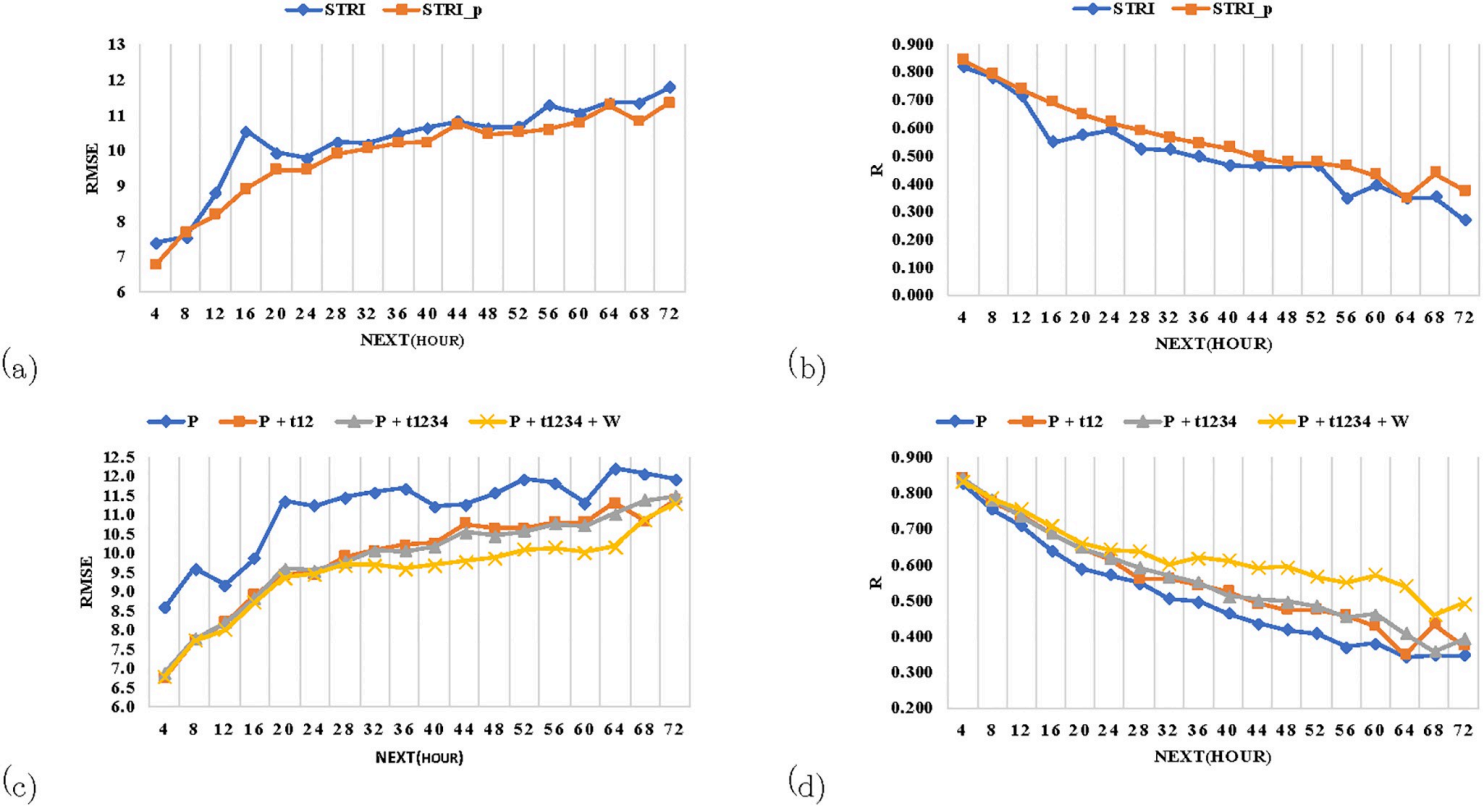
STRI, STRI_p and RTP performance. Top:Performance of STRI and STRI_p components from +4h to +72h in (a)RMSE and (b)R. Down:Results in (c)RMSE and (d)R given remote pollutant and local features using STRI_p component.

The effect of training strategies on prediction performance was evaluated by comparing the results from a full STRI model with those using the STRI_fe and STRI_p components, as described. STRI_p yields better prediction results than the full STRI model in both RMSE and R (Fig 7a and 7b). Since training a full STRI model on a single GPU can be challenging, STRI_fe for feature extraction and STRI_p for prediction were used. As STRI_p consists of a small number of layers, it converges quickly during training, leaving more room for model fine-tuning. Thus, the improved performance of STRI_p validates our idea of breaking the full STRI model into two components.

Secondly, the effects of the extracted remote pollutants and local features on the STRI_p component were evaluated. Experiments were conducted using one feature and incrementally added features while observing the results in terms of RMSE and R. Fig 7c and 7d shows the results of various features, including spatio-temporal features from two and four tiles (t12 and t1234) as well as the local PM_2.5_ (P) and weather (W) features from 18 stations. Therefore, the model input sequence is P, t12(tiles h28v06 and h29v06), the remaining two tiles(t34)(tiles h28v05 and h29v05) and then W.

A significant gap between the performance in R and RMSE when using P and when using data on remote pollutants from tiles t12 (P+t12) and t1234 (P+t1234) was observed (Fig 7(c) and 7(d)). Also the impact of expanding the range from tiles t12 to t1234 was observed. This impact is not present between +4h and +24h, possibly as events from t34 require additional time to make an impact. This fits with our goal of expanding the range to four tiles to improve prediction by capturing more RTPEs. The gap was attributed to long-term rather than short-term (+4h to +24h) weather fluctuations. Generally, the results show that the STRI_fe component captures knowledge from RTPEs by learning spatio-temporal behavior from AOD data with their corresponding weather features from remote areas.

**Fig 8:**
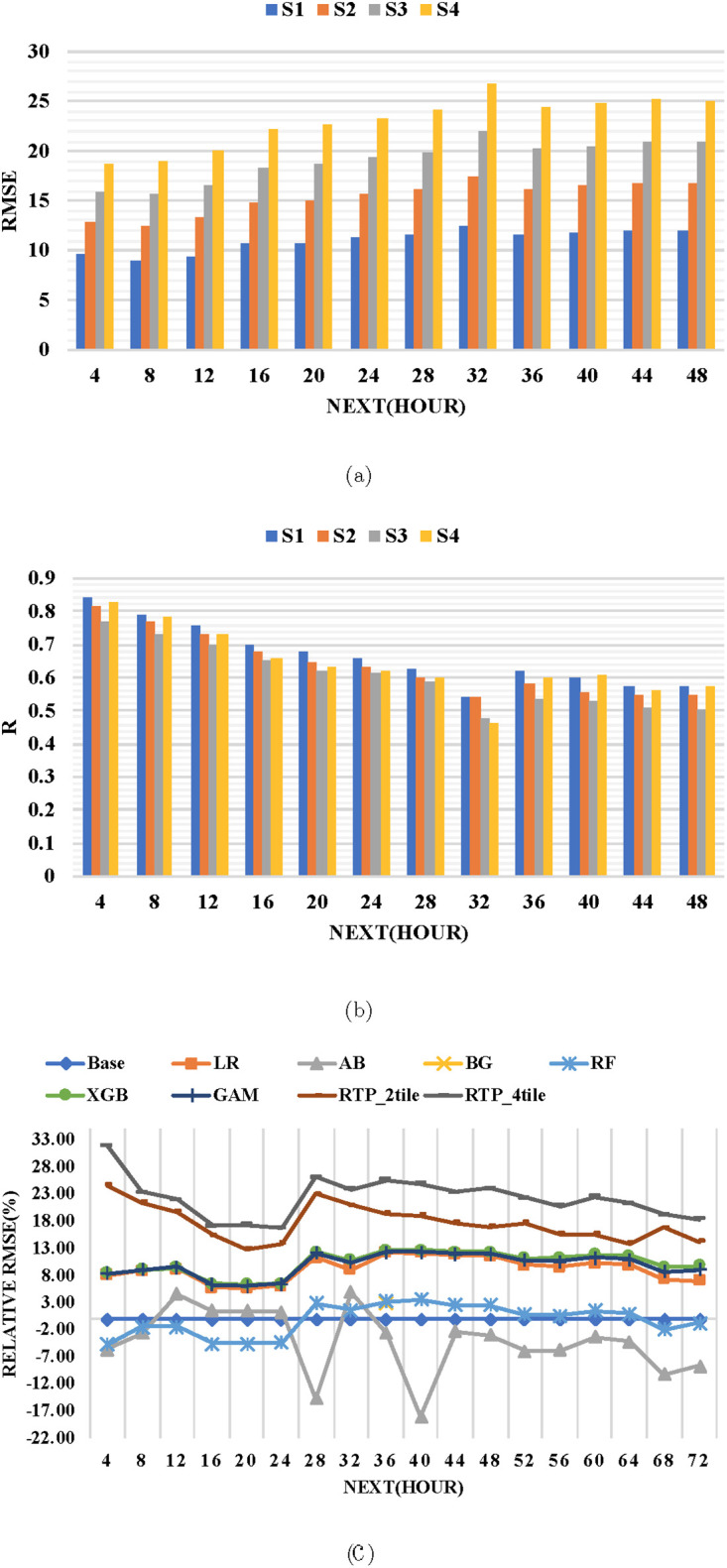
RTP model performance. The RTP performance in (a)RMSE and (b)R on four seasons in year. (c):Relative RMSE Improvement (%) of all models with reference to base model.

Thirdly, we evaluated the RTP model performance for four seasons in a year using prediction results of +4hr up to +48hr. We divided the dataset into four periods each having three months and evaluate the prediction results using RMSE and R. Season one (S1) starts from January to March, season two (S2) covers April to June, Season three (S3) starts July to September and season four (S4) covers the remaining months (October to December). Fig 8a and 8b shows RTP produces better performance in RMSE and R for S1 than all seasons in every prediction hour. Furthermore, S1 is the period where winter is at its peak and is the same period where northeasterly winter monsoon wind transport pollutants from central and northeastern Asia to Taiwan [45]. Therefore the better performance of RTP in S1 is probably contributed by the existence of a high level of pollutants in that period in the training datasets. In addition, S2 and S3 represent the spring and summer seasons which normally have a low levels of PM_2.5_ this reflects the performance of the RTP model in that period. S4 is the period when winter starts and expecting the level of PM_2.5_ to increase however the RTP performance does not imitate that scenario. Overall, the RTP performance matches with season variation with the level of PM_2.5_ in the northern part of Taiwan.

Fourth, we evaluated the performance of the RTP model in comparison with the Base model [27] and other state-of-the-art ensemble models with the same settings: linear regression (LR) [46], AB [32], BG, RF [34, 35], XGB [36–38], and a GAM [33, 39]. Note XGB yields better performance than gradient boosting machine [47] because of using more regularized model formalization to control over-fitting [48]. We also show RTP performance when we use RTPEs from 2tile (RTP_2tile) and 4tile (RTP_4tile) to show the impact of remote pollutants on the on the local prediction of PM_2.5_.

Fig 8(c) shows the relative prediction improvements in RMSE of both RTP models and the other state-of-the-art models w.r.t. Base model from +4h to +72h; the greater the improvement, the better the model does in comparison to the Base model. The figure shows that RTP_4tile yields the greatest improvements: from 17%–30%, 23%–26%, and 18%–22% for +4h to +24h, +28h to +48h, and +52h to +72h, respectively. Similarly, the RTP_2tile provides greater improvement: from 13%-24%, 17%-23%, and 13%-17% for +4h to +24h, +28h to +48h, and +52h to +72h. XGB and GAM, in turn, improve on the Base model by 6%–8%, 10%–12%, and 8%–11% for +4h to +24h, +28h to +48h, and +52h to +72h, respectively, with scores that are similar to those for the LR model. AB is outperformed by the Base model for most hours; RF is also, but to a lesser extent. The RTP_2tile and RTP_4tile outperform other models due to their composite neural network design [27], which involves high flexibility with learning capability to model nonlinear association between input features. The performance of RTP_4tile over RTP_2tile continues to demonstrate the importance of the enlarged remote area to capture more RTPEs from the remote area.

## Conclusion

To characterize the occurrence of remote transportation pollution events (RTPEs), we define it as a combination of thresholds and increments in one hour of PM_2.5_ concentration, and then design an algorithm to classify PM_2.5_ concentrations into RTPEs. The proposed RTPE and algorithm are evaluated for the area in northern Taiwan and the corresponding satellite data, and we believe that the proposed method can be applied elsewhere. In particular, the evaluation shows that a well-designed deep learning model extracts the knowledge from satellite data that it improves the accuracy of PM_2.5_ prediction.

It is worth noting that RTPEs can be captured using the proposed composite RTP model, and then RTPEs can be aqpplied to improve the prediction of PM_2.5_. The proposed RTP comprises two main components: a pre-trained Base model and the STRI model that capture the knowledge of local PM_2.5_ concentrations and RTPEs, respectively. In addition, STRI learns spatio-temporal characteristics of AOD data and weather features through its component STRI_fe, and then predicts local PM_2.5_ through the other component STRI_p that their performances are validated from empirical study. Local PM_2.5_ predictions using the RTP model outperform the base model and other state-of-the-art ensemble models by 12%–30%, 12%–18%, and 10%–14% for +4h to +24h, +28h to +48h, and +52h to +72h, respectively. The ESD model, although it only considers local AOD data, still improves PM_2.5_ prediction, as evidenced by lower RMSE scores than the base model by 12.68% for +1day and 11.45% and 6.65% for +2day and +3day.

The outstanding performance of the STRI model on the prediction of PM_2.5_ will help the government policy maker to take measures, including controlling traffic in the area that is expected to have a high level of PM_2.5_. They may also use that information for warning systems and plan mitigation actions to reduce the risk to public health. In addition, the same information can be used by individuals to organize their activities, such as whether to exercise outside.

Future work will focus on expanding the remote area, using data that are updated at a higher frequency compared to AOD data, and considering other possible features and models.

## Supporting information

S1 Table STRI model configuration details. (PDF)

S1 Appendix More details on classifying remote pollutants. (PDF)
