# Supplementary material for: Using satellite data on remote transportation of air pollutants for PM2.5 prediction in northern Taiwan
Source: PLoS One. 2023 Mar 10;18(3):e0282471. doi: 10.1371/journal.pone.0282471 (PMC10004525; doi:10.1371/journal.pone.0282471)
Supplement: S1 Table — (PDF) [file pone.0282471.s001.pdf]

TABLE 1:
DETAIL CONFIGURATION FOR STRI_fe

**Tile 1**	**Tile 2**	**Tile 3**	**Tile 4**	**Remote Weather**
**Layer Name**	**Layer Name**	**Layer Name**	**Layer Name**	**Layer Name**
Avg-pooling 3D 1x2x2 stride 1x2x2 padding same	Avg-pooling 3D 1x2x2 stride 1x2x2 padding same	Avg-pooling 3D 1x2x2 stride 1x2x2 padding same	Avg-pooling 3D 1x2x2 stride 1x2x2 padding same	ConvLSTM2D 1x3 64 units ReLU
Conv2D 3x3 32 maps ReLU	Conv2D 3x3 32 maps ReLU	Conv2D 3x3 32 maps ReLU	Conv2D 3x3 32 maps ReLU	BatchNormalization layer
Conv2D 3x3 32 maps ReLU	Conv2D 3x3 32 maps ReLU	Conv2D 3x3 32 maps ReLU	Conv2D 3x3 32 maps ReLU	ConvLSTM2D 1x3 32 units ReLU
Dropout rate 0.3	Dropout rate 0.3	Dropout rate 0.3	Dropout rate 0.3	-
ConvLSTM2D 3x3 32 units ReLU	ConvLSTM2D 3x3 32 units ReLU	ConvLSTM2D 3x3 32 units ReLU	ConvLSTM2D 3x3 32 units ReLU	-
BatchNormalization layer	BatchNormalization layer	BatchNormalization layer	BatchNormalization layer	-
ConvLSTM2D 3x3 32 units ReLU	ConvLSTM2D 3x3 32 units ReLU	ConvLSTM2D 3x3 32 units ReLU	ConvLSTM2D 3x3 32 units ReLU	-
Avg-pooling 1x10x10 stride 1x10x10 padding same	Avg-pooling 1x10x10 stride 1x10x10 padding same	Avg-pooling 1x10x10 stride 1x10x10 padding same	Avg-pooling 1x10x10 stride 1x10x10 padding same	-
Flatten	Flatten	Flatten	Flatten	Flatten
Concatenate				
**Repeat vector (output is saved as ERP)**				

TABLE 2:
DETAIL CONFIGURATION FOR STRI_p

**Local Weather**	**Local PM2.5**	**ERP**
**Layer Name**	**Layer Name**	**Layer Name**
Repeat vector	Repeat vector	-
Concatenate		
Fully connected 32 Units ReLU		
Dropout rate 0.3		
Fully connected 32 Units ReLU		
Dropout(rate 0.4)		
Fully connected 18 Units linear		
