# Supplementary material for: Using satellite data on remote transportation of air pollutants for PM2.5 prediction in northern Taiwan
Source: PLoS One. 2023 Mar 10;18(3):e0282471. doi: 10.1371/journal.pone.0282471 (PMC10004525; doi:10.1371/journal.pone.0282471)
Supplement: S1 Appendix — (PDF) [file pone.0282471.s002.pdf]

Remote Pollutants Classification Details

The numerical prediction score of $PM_{2.5}$ is converted into decision by checking if the score is above the defined threshold values. Base on the International Organization for Standardization (ISO) 5725, numeric accuracy is the decomposition of numerical quantities into numerical version of trueness and precision. Therefore, we calculate the score of the evaluation metrics including accuracy, precision, recall and F1_score between prediction results and true values using three defined thresholds of $PM_{2.5}$. We use the confusion matrix as a tool to calculate those metrics, the confusion matrix contains all the information that can be used to analyze the errors and confusion of the end results Furthermore, the confusion matrix consists of true positive (TP) information which in this work we define as predicted $PM_{2.5}$ value which satisfy both thresholds condition. The matrix also contains true negative (TN), false positive (FP) and false negative (FN) information, Table 1 shows the details of the remote transportation event confusion matrix that match with this task. The equations below shows how to calculate all classification metrics.

Given $PM_{2.5}$ ground truth(GT) values and their predicted values as remote transportation (RT) with their corresponding first order differential vector GTD and RTD. Moreover given the Epa_tshd(β_1), Diff_tshd(β_2) and the total number of hours that used in prediction(all hours in winter and autumn seasons). We obtain four events(E) after applying those thresholds to $PM_{2.5}$ values.
E_1:GT$> \beta_1$, E_2:GTD$> \beta_2$, E_3:RT$> \beta_1$, E_4:RTD $> \beta_2$
Then we use probability(P) to obtains the number of remote transportation pollution events by considering their occurrence with total number of hours(t). Later, the confusion matrix in Table 1 shows how to calculate true positive(TP), false negative(FN), false positive(FP), and true negative(TN), which show the full picture of our model performance.

$$P(GT) = \frac{count\{E_1\}}{total\{t\}} \tag{1}$$

$$P(GT') = \frac{count\{E_2\}}{total\{t\}} \tag{2}$$

$$P(GT|GT') = \frac{count\{E_1 \cap E_2\}}{E_2} \tag{3}$$

$$P(GT \cap GT') = \frac{count\{E_1 \cap E_2\}}{total\{t\}} == P(GT|GT')P(GT') \tag{4}$$

$$P(RT) = \frac{count\{E_3\}}{total\{t\}} \tag{5}$$

$$P(RT') = \frac{count\{E_4\}}{total\{t\}} \tag{6}$$

$$P(RT|RT') = \frac{count\{E_3 \cap E_4\}}{E_4} \tag{7}$$

$$P(RT \cap RT') = \frac{count\{E_3 \cap E_4\}}{total\{t\}} == P(RT|RT')P(RT') \quad (8)$$

Table 1: CONFUSION MATRIX OF REMOTE EVENT

		Predicted $PM_{2.5}$ values	
		Remote event=Yes	Remote event=No
Ground Truth $PM_{2.5}$	Remote Event=Yes	TP= $(GT \cap GT') \cap (RT \cap RT')$	FN= $(GT \cap GT') \cap \neg(RT \cap RT')$
	Remote event=No	FP= $\neg(GT \cap GT) \cap (RT \cap RT')$	TN= $\neg(GT \cap GT') \cap \neg(RT \cap RT')$
